# Characterization of the ATP-Dependent Lon-Like Protease in *Methanobrevibacter smithii*

**DOI:** 10.1155/2016/5759765

**Published:** 2016-04-28

**Authors:** Jihua Pei, Jianfang Yan, Yi Jiang

**Affiliations:** ^1^Department of Gastroenterology, The Second Affiliated Hospital of Wenzhou Medical University, Wenzhou 325027, China; ^2^Plant Protection College, Shenyang Agricultural University, Shenyang 110161, China; ^3^College of Life Science, Dalian Nationalities University, Dalian 116600, China

## Abstract

The Lon protease is highly evolutionarily conserved. However, little is known about Lon in the context of gut microbial communities. A gene encoding a Lon-like protease (Lon-like-Ms) was identified and characterized from* Methanobrevibacter smithii*, the predominant archaeon in the human gut ecosystem. Phylogenetic and sequence analyses showed that Lon-like-Ms and its homologs are newly identified members of the Lon family. A recombinant form of the enzyme was purified by affinity chromatography, and its catalytic properties were examined. Recombinant Lon-like-Ms exhibited ATPase activity and cleavage activity toward fluorogenic peptides and casein. The peptidase activity of Lon-like-Ms relied strictly on Mg^2+^ (or other divalent cations) and ATP. These results highlight a new type of Lon-like protease that differs from its bacterial counterpart.

## 1. Introduction

The ATP-dependent Lon (La) protease is the most highly conserved member of the energy-dependent protease present in the cytosol of prokaryotes and in the mitochondria and peroxisomes of eukaryotes [1–3]. Lon protease derives its name from the phenotype of* Escherichia coli *Lon gene mutants, which form long, undivided filaments upon UV irradiation [4]. Lon protease is an essential component of the protein quality-control systems that have evolved in all cells to protect against the harmful effects of unfolded proteins. Key components of these systems include ATP-dependent proteases, chaperones, heat-shock proteins, and regulatory molecules [5]. ATP-dependent proteases belonging to the AAA protein superfamily (ATPases associated with diverse cellular activities), including the 26S proteasome, HslUV, and Clp complexes, comprise separate subunits for ATP hydrolysis and proteolysis; in contrast, Lon is a homooligomer composed of identical subunits that include both ATPase and protease domains. To avoid unnecessary degradation of cellular proteins, substrate selection by quality-control proteases is tightly regulated.* Escherichia coli* Lon can recognize specific aromatic residue-rich sequences that are accessible in unfolded polypeptides but hidden in the most native structures. Lon also unfolds and degrades stably folded proteins with accessible recognition tags [6].

Two subfamilies of Lon proteases, LonA and LonB, were defined based on sequence analysis [7]. The LonA subfamily mainly comprises bacterial and eukaryotic enzymes that mimic the classical Lon protease from* E. coli*. The LonB subfamily proteins are only found in Archaea, which is contradictory to the detection of LonB-like proteases in bacteria. The LonB protease with membrane-bound property was firstly isolated from* Thermococcus kodakarensis* [8]. LonB proteases may have the same functions of the only bacterial membrane-bound ATP-dependent protease FtsH, which is not present in Archaea. Many archaeal LonB homologs have been characterized, including* T. kodakarensis* TK1264 (*Tk*LonB) [8],* Thermoplasma acidophilum* Ta1081 (*Ta*LonB) [9],* Methanocaldococcus jannaschii* MJ1417 (*Mj*LonB) [10],* Archaeoglobus fulgidus* AF0364 (*Af*LonB) [11], and* Haloferax volcanii* HVO_0783 (*Hv*LonB) [12]. All LonB proteases have a similar domain organization, which includes an N-terminal AAA domain with two transmembrane-spanning helices and a C-terminal protease domain. Biochemical studies of recombinant LonB have been limited to proteins purified from* E. coli*, such as full-length* Tk*LonB,* Ta*LonB, and* Af*LonB, as well as the protease domains of* Af*LonB and* Mj*LonB. However, some Archaea contain both LonA and LonB proteases, such as* Methanosarcinaceae.* Several bacterial genomes including* E. coli*,* Thermotoga maritima*, and* Vibrio cholerae* encode LonA proteases and LonB-like proteases. In some Archaea or* Caenorhabditis elegans*, some Lon-like proteases that cannot be characterized clearly belonging to either the LonA or LonB subfamilies have also been identified [7].

*Methanobrevibacter smithii* is the predominant archaeon in the human gut ecosystem [13]. This organism plays an important role in the efficient digestion of polysaccharides (complex sugars) by consuming the end products of bacterial fermentation [14].* M. smithii* may thus be a therapeutic target for reducing energy harvesting in obese humans, and metagenomic studies of the gut microbial communities in genetically obese mice and lean littermates have shown that the former exhibit an enhanced representation of the genes involved in polysaccharide degradation, possess more Archaea, and possess a greater capacity to promote adiposity when transplanted into germ-free recipients [13]. Despite its importance, little is known about the function of Lon in* M. smithii*. Two genes (Msm_1569 and Msm_1754) have been annotated as Lon in the genome sequence, and the encoded proteins may play different roles. Msm_1569 encodes a canonical LonB protease (Lon-Ms). In this study, we provide the first description of the sequence characteristics of Msm_1754 (Lon-like-Ms), which differs considerably from previously reported Lon proteases. Furthermore, we expressed and purified the soluble recombinant form of Lon-like-Ms from* E. coli* and biochemically analyzed its enzymatic properties.

## 2. Materials and Methods

### 2.1. Protein Expression and Purification

*M. smithii* strain PS (ATCC 35061) was cultivated in 125 mL serum bottles containing 15 mL of MBC medium supplemented with 3 g/L formate, 3 g/L acetate, and 0.3 mL of a freshly prepared, anaerobic, filter-sterilized 2.5% Na_2_S solution. The remaining volume in the bottle (headspace) contained a 4 : 1 mixture of H_2_ and CO_2_; the headspace was replenished every 1-2 days during a 6-day growth period at 37°C. DNA was recovered from harvested cell pellets using the Qiagen Genomic DNA Isolation kit (Valencia, CA, USA), with mutanolysin (1 unit/mg wet-weight cell pellet; Sigma, St. Louis, MO, USA) added to facilitate microbe lysis.

*M. smithii* genomic DNA was used as a template in a polymerase chain reaction (PCR), which isolated* Lon-like-Ms* (Msm_1754 and WP_011954752) using the following oligonucleotide primers: forward, 5′-GGA ATT CGA AGA ACC AAA GCC GCT GAAC-3′ and reverse, 5′-CCC AAG CTT TCA CCA CCG CCG GTG C-3′. PCR products were ligated into the pET28a vector and sequenced before transformation into BL21 (DE3).* Escherichia coli* BL21 (DE3) cells containing the* pET28-Lon-like-Ms* plasmid were cultured. When the OD_600_ reached 0.7, isopropyl-*β*-d-thiogalactopyranoside (IPTG) was added to induce protein expression. The cells were cultured in the presence of IPTG for 4 h with shaking and then harvested and resuspended in lysis buffer containing 50 mM Tris (pH 8.0), 300 mM NaCl, 20 mM 2-mercaptoethanol, and 20 mM imidazole. The cell suspension was sonicated and centrifuged, and the supernatant was loaded on a Ni-NTA column. After washing the column with lysis buffer, Lon-like-Ms was eluted using an imidazole gradient (50–250 mM). Purified Lon was separated on a 10% sodium dodecyl sulfate polyacrylamide gel electrophoresis (SDS-PAGE) and visualized. Protein concentrations were estimated using the Bradford method and bovine serum albumin (BSA) as a standard [15].

### 2.2. Enzyme Activity Assays

Peptide cleavage activity was assayed using the fluorogenic peptides glutaryl-Ala-Ala-Phe-4-methoxy-*β*-naphthylamide (Glt-AAF-MNA) and succinyl-Phe-Leu-Phe-4-methoxy-*β*-naphthylamide (Bachem, Bubendorf, Switzerland) [16]. The reaction mixture comprised 0.3 mM fluorogenic peptide, 1 or 4 mM ATP, 10 mM MgCl_2_, and 5 *μ*g Lon-like-Ms in 500 *μ*L of 50 mM Tris buffer (pH 8.0). The mixture was incubated at 37°C for 6 min, and the increase in fluorescence (excitation, 350 nm; emission, 440 nm) was monitored using a spectrofluorometer (model F-2000; Hitachi, Tokyo, Japan).

The proteolytic activity of the purified protein was determined using *α*-casein as substrate. A 500 *μ*L reaction volume was prepared in a microfuge tube containing purified Lon-like-Ms (5 *μ*g), *α*-casein (300 *μ*g) dissolved in 50 mM Tris-HCl (pH 8.0), and the indicated amounts of other reagents. After incubation at 37°C for 6 min, the reaction was stopped by adding 50 *μ*L of 50% trichloroacetic acid to precipitate the unreacted substrates. After centrifugation to remove the precipitates (12,000 g, 10 min), the enzymatic products in the supernatant were quantified at a wavelength of 280 nm using a UV-visible spectrophotometer (Shimadzu, Japan).

ATPase activity was assayed by determining the amount of P_i_ liberated from ATP. Lon-like-Ms (2.5 *μ*g) was incubated with 4 mM ATP and 10 mM MgCl_2_ in 100 *μ*L of 50 mM Tris buffer (pH 8.0) at 37°C for 15 min. Free P_i_ was determined according to the procedure described by Black and Jones [17]. Background levels of hydrolysis (no enzyme) were subtracted in each assay.

## 3. Results

### 3.1. Sequence Analysis of Lon-Like-Ms

**Figure 1:**
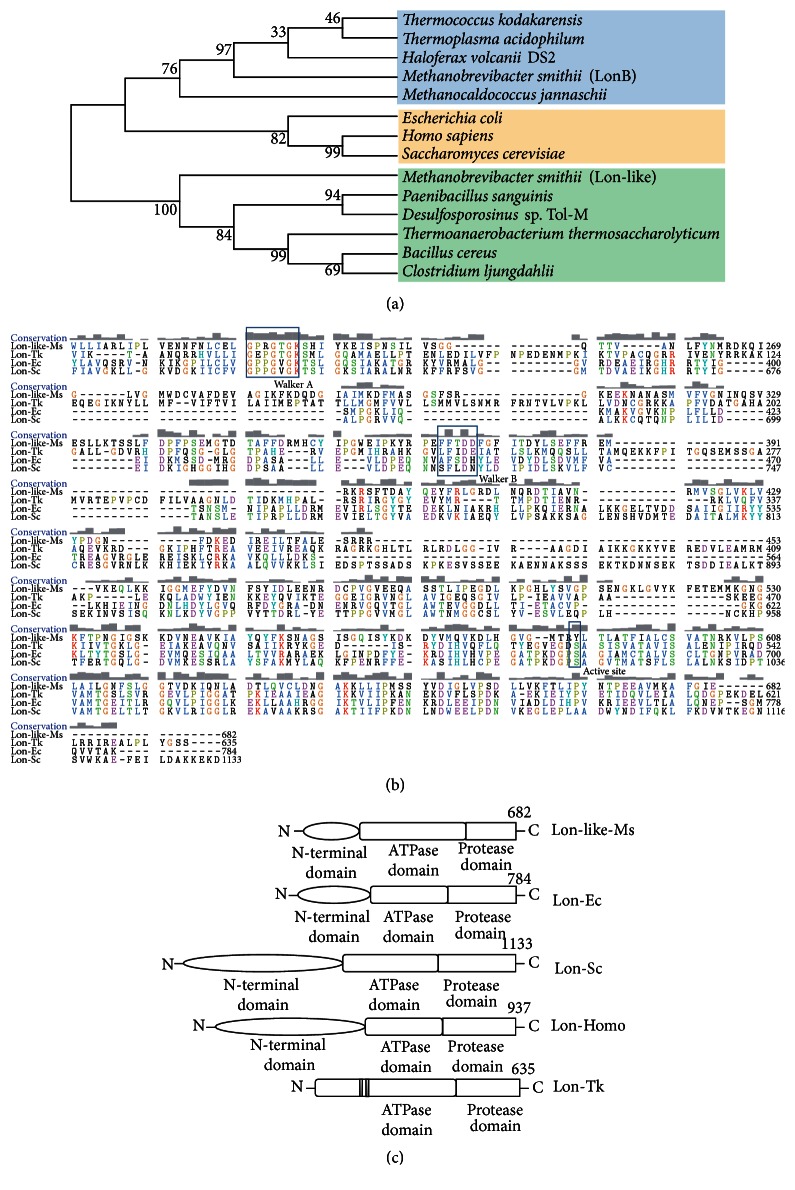
Phylogenetic and sequence analysis of Lon-like-Ms. (a) Unrooted neighbor-joining phylogenetic tree of Lon from the LonA (yellow), LonB (blue), and Lon-like (green) families, generated using MEGA5. The optimal tree with a branch length sum of 13.47550492 is shown. The percentage of replicate trees in which the associated taxa clustered together in the bootstrap test (1000 replicates) is shown next to the branches. (b) Lon-like-Ms and protease Lon sequences from* T. kodakarensis* KOD1,* E. coli*, and yeast were aligned. The Walker A, Walker B, and the possible active site are shown in the rectangles. The conservation level of each residue is indicated by the height of the bars above each residue. The number at the ending of each line of amino acids indicates the number of the amino acid residues. (c) Putative domains of Lon proteases from* M. smithii* (Lon-like-Ms and WP_011954752),* E. coli* (Lon-Ec, WP_001295325.1), yeast (Lon-Sc, NP_009531.1), human (Lon-Homo, NP_004784.2), and* T. kodakarensis* (Lon-Tk, WP_011250215.1).

A National Center for Biotechnology Information (NCBI) BLAST-P search for the Lon-like-Ms sequence revealed the most significant homology (35–64% identity) with the* Paenibacillus*,* Desulfosporosinus*,* Thermoanaerobacterium*,* Bacillus*, and* Clostridium* families. To explore the evolutionary relationship between Lon-like-Ms and other annotated Lons, the MEGA5 program was used to construct a phylogenetic tree from amino acid sequence data of previously studied Lon members from the LonA (human, yeast,* E. coli*) and LonB (some extremophiles) subfamilies (Figure 1(a)). Although the bootstrap values were somewhat low because of a large number of sequences, more significant bootstrap values in the distal branches allowed us to infer those proteins from similar species which were derived from a common ancestor. Furthermore, their positions in the dendrogram were independent of the method used for phylogenetic reconstruction (data not shown). These clustering results suggest that Lon-Ms protease is located within the same cluster as LonB from other Archaea. However, Lon-like-Ms protease and its homologs form a cluster distinct from LonA and LonB. The microorganisms in this cluster, which include* Paenibacillus*,* Desulfosporosinus*,* Thermoanaerobacterium*,* Bacillus*, and* Clostridium*, are strictly or facultatively anaerobic. Accordingly, this group of Lon proteases was tentatively assigned the designation of Lon-like protease. A ClustalW sequence alignment (http://www.ebi.ac.uk/Tools/msa/clustalw2/) also found consistently high identity (>35%) among Lon proteases from the same subfamily, in contrast to low identity (<20%) of Lon-like-Ms and its homologs from* Paenibacillus*,* Desulfosporosinus*,* Thermoanaerobacterium*,* Bacillus*, and* Clostridium* with proteins from the LonA and LonB subfamilies (Supplementary Table 1 in Supplementary Material available online at http://dx.doi.org/10.1155/2016/5759765). The detailed sequence alignment (Figure 1(b)) showed that Lon-like-Ms contains the conserved Walker A and Walker B motifs in the N-terminal of Lon-like-Ms sequence. Interestingly, the amino acids Tyr587 of Lon-like-Ms correspond to the highly conserved catalytic serine residue in LonA and LonB subfamily.

The enzyme domains were further analyzed using the Simple Modular Architecture Research Tool (SMART) (http://smart.embl-heidelberg.de/) (Figure 1(c)). The results revealed that all of them share an ATPase domain and a C-terminal protease domain. Lon-like-Ms contains another N-terminal domain like LonB. The transmembrane regions of Lon-like-Ms and Lon from* E. coli* (LonA) and* T. kodakarensis* (LonB) were predicted using TMHMM v.2.0 (http://www.cbs.dtu.dk/services/TMHMM/) and TOPCONS (http://topcons.cbr.su.se/). In contrast to LonA, the LonB subfamily contains two transmembrane helices in the ATPase domain. Interestingly, no transmembrane motif was observed in Lon-like-Ms and other members of the Lon-like subfamily (Supplementary Figure 1).

### 3.2. Expression and Purification of Lon-Like-Ms

**Figure 2:**
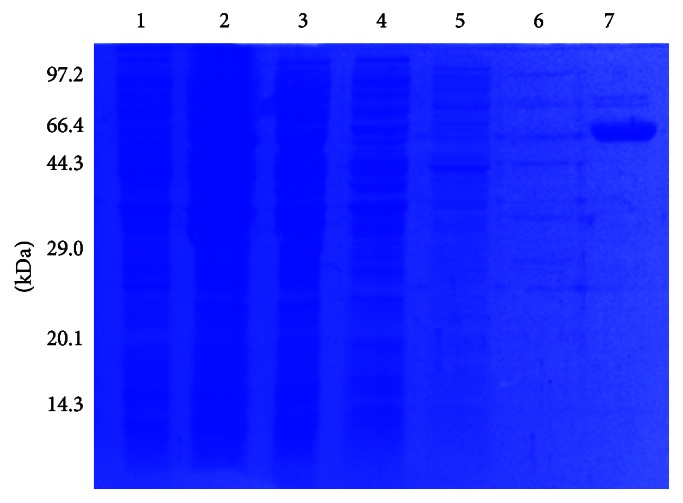
Expression and purification of Lon-like-Ms from* E. coli*. Molecular mass standards are indicated at left. Lane 1, crude protein extract from noninduced cells; Lane 2, crude protein extract from IPTG-induced cells; Lane 3, soluble extract from IPTG-induced cells; Lane 4, unbound proteins eluted from the Ni-NTA column; Lane 5, proteins eluted with 5 mM imidazole; Lane 6, proteins eluted with 10 mM imidazole; Lane 7, proteins eluted with 300 mM imidazole.

To study the function of Lon-like-Ms, we first expressed the encoding gene in a bacterial system. The gene encoding Lon-like-Ms was amplified via PCR from* M. smithii* genomic DNA. The gene fragment was verified by size following agarose gel electrophoresis and was subcloned into the* E. coli* expression vector pET28a. The inserted fragment was confirmed by DNA sequencing.* E. coli* was transformed with the* pET28a-Lon-like-Ms* vector and cultured, after which protein expression was induced with 1-mM IPTG. Bacterial samples were collected and analyzed by SDS-PAGE (Figure 2). As indicated in lane 3, a protein band with a molecular weight of approximately 77 kDa, which matched the predicted molecular mass (77.4 kDa) of Lon-like-Ms, was detected after IPTG induction. After cultivation,* E. coli *cells were resuspended in the required buffer and sonicated. After centrifugation, recombinant Lon-like-Ms was effectively purified by Ni-NTA affinity chromatography, as indicated by a single band on the SDS-PAGE gel (Figure 2).

### 3.3. Lon-Like-Ms Activity Assay

**Figure 3:**
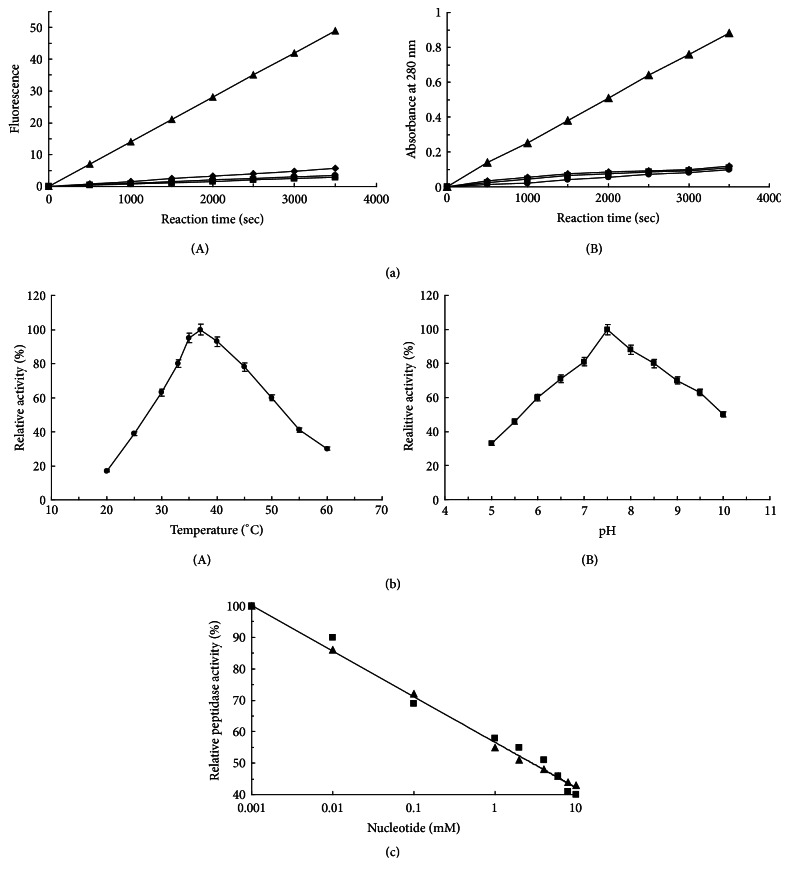
Lon-like-Ms activity assay. (a) Time courses of fluorogenic peptide (A) and *α*-casein (B) hydrolysis by Lon-like-Ms. Reactions were conducted in 50 mM Tris-HCl buffer (pH 8.0) containing 5 *μ*g of Lon-like-Ms and 0.3 mM Glt-AAF-MNA at 37°C. ATP and/or MgCl_2_ were added to the mixture as follows: 4 mM ATP and 10 mM MgCl_2_ (filled triangle); no addition (filled quadrangle); 10 mM MgCl_2_ (filled cycle); or 4 mM ATP (filled diamond). (b) Effects of temperature (A) and pH (B) on the peptide cleavage activity of Lon-like-Ms. Glt-AAF-MNA was used as a substrate for the peptide cleavage assay. (c) Effects of ATP (filled triangle) and AMP-PNP (filled quadrangle) concentrations on the peptide cleavage activity of Lon-like-Ms.

The peptide cleavage activity of Lon-like-Ms was determined using Glt-AAF-MNA, which has often been used as a substrate for various Lon proteases. When Lon-like-Ms was incubated with Glt-AAF-MNA in the presence of ATP and Mg^2+^, a time-dependent increase in fluorescence, consequent to the hydrolysis of the fluorogenic peptide, was observed (Figure 3(a)). Lon-like-Ms exhibited no cleavage activity without Mg^2+^, regardless of the presence or absence of ATP. A similar result was observed when *α*-casein was used as a substrate for proteolytic activity analysis. The optimum pH and temperature for ATP-independent Lon-like-Ms activity were found to be 7.5 and 37°C (Figure 3(b)), respectively. Under these optimal conditions, Lon-like-Ms exhibited a specific activity of 34 ± 8.7 pmol of MNA/*μ*g of protein/h toward Glt-AAF-MNA in the presence of 4-mM ATP.

Lon-like-Ms exhibited peptidase and protease activity in the presence of various nucleoside triphosphates and divalent cations, as shown in Table 1. ADP, a potent inhibitor of Lon from* E. coli*, also inhibited the activity of Lon-like-Ms, although the enzyme retained 64% activity relative to the level detected in the presence of ATP. Activity in the presence of a nonhydrolyzable analog of ATP, 5′-adenylyl imidodiphosphate (AMP-PNP), was similar to that observed with ATP, as observed for Lon from* E. coli*. An increase in the ATP or AMP-PNP concentration resulted in a decrease in activity, reaching a decrease by half at 1-mM ATP or AMP-PNP (Figure 3(c)). These results suggested that, for Lon-like-Ms, inhibition via nucleotide binding was quite distinct from that for Lon from* E. coli*, as the activity of the latter increased with an increase in the ATP concentration from 10^−4^ to 10^−2^ mM. Enzymatic activity was strictly dependent on the presence of divalent cations, such as Mg^2+^. Interestingly, Mn^2+^ behaved similarly to Mg^2+^ as a cofactor, and Ca^2+^ could replace Mg^2+^ to a similar extent. Although less effective than Mg^2+^, Ni^2+^, or Ca^2+^, Co^2+^ was also able to support hydrolysis (Table 1). The rates of Lon-like-Ms-mediated hydrolysis of different nucleotides were also measured (Table 2). Activity was found to be strictly dependent on divalent cations, such as Mg^2+^, and other divalent cations (Ni^2+^, Ca^2+^, Mn^2+^, and Co^2+^) supported activity levels of 52–105% relative to that observed with Mg^2+^ (Table 2).

## 4. Discussion and Conclusion

To our knowledge, Lon-like-Ms is the first studied Lon-like protease expressed in an archaeon from the human gut. Similar to other LonA proteases from various sources, Lon-like-Ms possesses a three-domain structure comprising the N-terminal domain, ATPase domain, and protease domain. Although Lon-like-Ms exhibited reduced similarity to its bacterial and eukaryotic counterparts, it was classified as a member of the AAA superfamily and was found to possess several conserved motifs, such as the nucleotide-binding Walker A and Walker B [18]. Two subfamilies of Lon proteases, LonA and LonB, have previously been classified based on consensus sequences at proteolytic sites in the protease domains [7]. In general, these two subfamilies are associated with two distinct Walker motif consensus sequences in the AAA domains and specific domain organization features wherein an N-terminal domain is attached to the AAA modules of all LonA but not LonB members. The latter members are found only in Archaea, and their AAA domains possess additional integrated membrane-spanning segments that anchor the proteins to the membrane [8]. However, this proteolytic site-based classification approach excluded a large group of Lon-like proteases encoded in the genomes of many gram-negative bacteria (as well as certain gram-positive bacteria and Archaea) that contain LonB-like consensus sequences at the proteolytic sites but lack transmembrane regions or detectable AAA consensus motifs [19]. Even though Lon-like-Ms contains both an ATPase domain and proteolytic domain, it exhibited low sequence similarity to both LonA and LonB. Lon-like-Ms also lacks a transmembrane region and thus differs from LonB proteases in Archaea. Furthermore, LonA and LonB use serine as an active site because the hydroxyl group of serine is able to act as a nucleophile, attacking the carbonyl carbon of the scissile peptide bond of the substrate. In the sequence alignment (Figure 1(b)), tyrosine occupies the position of serine in Lon-like-Ms. Tyrosine, an aromatic amino acid with hydroxyl group, can also be a nucleophile (as in DNA topoisomerases) [20]. Lon-like-Ms may also use other serines in the C-termini as the active sites because the folding of catalytic domain of Lon-like-Ms is not clear. Taken together, these studies demonstrate that Lon-like-Ms and its homologs form a novel family that is distinct from both the LonA and LonB families.

In the presence of ATP, Lon cleaves protein substrates at multiple sites into small peptide products. During this process, Lon hydrolyzes ATP, unfolds and/or translocates protein substrates to the proteolytic active site, and catalyzes peptide bond cleavage. When analyzing the kinetic mechanism underlying the Lon-mediated degradation of protein substrates, it is difficult to determine the specific functions of ATP binding and ATP hydrolysis during each ATPase cycle because the substrate contains multiple and diverse sites that are recognized and/or cleaved by Lon [2]. In this study, we further purified and characterized Lon-like-Ms to address this biochemical issue. Lon-like-Ms exhibited activity against different nucleotides. This protease also exhibited cleavage activity against fluorogenic peptides in the presence of ATP and Mg^2+^. Lon from* E. coli* [21] exhibits maximal efficiency when ATP is bound and hydrolyzed in the presence of Mg^2+^. However, fluorogenic peptide degradation was also shown to occur in the presence of nonhydrolyzable AMP-PNP. Unexpectedly, Lon from* T. kodakarensis* [8] and* T. acidophilum* [9] exhibited higher peptide cleavage activity in the absence of ATP than in the presence of ATP. A similar Lon-like protein, TTC1975, from* Thermus thermophilus* was shown to lack ATPase activity, and thus its proteolytic activity was not stimulated by the addition of ATP [22]. These results indicate that archaeal Lon-like-Ms and bacterial Lon from* E. coli* are similar with regard to ATP dependency. Conversely, the inhibition of Lon-like-Ms via nucleotide binding was quite distinct from that of Lon from* E. coli*. Finally, archaeal Lon-like-Ms does not contain a transmembrane region, unlike the Lon proteases from* T. kodakarensis* and* T. acidophilum*, and therefore the sequence identities between Lon-like-Ms and these other proteins are very low. To further elucidate the function of Lon-like-Ms, we are currently conducting experiments to identify the substrates of Lon-like-Ms by constructing mutant strains in which Lon-like-Ms will be overexpressed.

## Supplementary Material

Supplementary Figure 1. Transmembrane prediction of Lon-like-Ms, Lon-Ec and Lon-Tk by TMHMM (A) and TOPCONS (B).
Supplementary Table 1. Percent sequence identity matrix of Lon-like and Lon proteases from *Methanobrevibacter smithii* (msm_1754 (WP_011954752) and msm_1569 (WP_011954598.1)), *Paenibacillus sanguinis* (WP_018751256.1), *Desulfosporosinus* sp. Tol-M (KGP77159.1), *Thermoanaerobacterium thermosaccharolyticum* (WP_013297045.1), *Bacillus cereus* (WP_001993072.1), *Clostridium ljungdahlii* (WP_013239896.1), *Escherichia coli* (WP_001295325.1), *Homo sapiens* (NP_004784.2), *Saccharomyces cerevisiae* (NP_009531.1), *Haloferax volcanii* (WP_004044154.1), *Thermococcus kodakarensis* (WP_011250215.1), *Thermoplasma acidophilum* (WP_010901491.1), and *Methanocaldococcus jannaschii* (AAB99427.1).

## Figures and Tables

**Table 1:** Relative cleavage activities of Lon-like-Ms in the presence of different nucleotides or divalent cations.

Nucleotide	Divalent cation	Peptide hydrolysis (%)	Casein hydrolysis (%)
None	None	5	7
ATP	Mg^2+^	100	100
ATP	None	6	4
None	Mg^2+^	10	8
AMP-PNP	Mg^2+^	92	80
ADP	Mg^2+^	64	72
GTP	Mg^2+^	90	85
CTP	Mg^2+^	131	122
UTP	Mg^2+^	64	55
ATP	Ni^2+^	20	16
ATP	Ca^2+^	83	80
ATP	Mn^2+^	89	72
ATP	Co^2+^	70	65
ATP	Zn^2+^	12	9

**Table 2:** Relative hydrolysis rates of different nucleotides by Lon-like-Ms and the effects of divalent cations.

Nucleotide	Divalent cation	Nucleotide hydrolysis (%)
ATP	None	0
ATP	Mg^2+^	100
AMP-PNP	Mg^2+^	84
ADP	Mg^2+^	64
AMP	Mg^2+^	6
GTP	Mg^2+^	78
CTP	Mg^2+^	105
UTP	Mg^2+^	58
ATP	Ni^2+^	52
ATP	Ca^2+^	66
ATP	Mn^2+^	105
ATP	Co^2+^	70
