# Supplementary material for: Characterization of the ATP-Dependent Lon-Like Protease in Methanobrevibacter smithii
Source: Archaea. 2016 Apr 28;2016:5759765. doi: 10.1155/2016/5759765 (PMC4864567; doi:10.1155/2016/5759765)
Supplement: Supplementary file 1 — Supplementary Figure 1. Transmembrane prediction of Lon-like-Ms, Lon-Ec and Lon-Tk by TMHMM (A) and TOPCONS (B). Supplementary Table 1. Percent sequence identity matrix of Lon-like and Lon proteases from Methanobrevibacter smithii (msm_1754 (WP_011954752) and msm_1569 (WP_011954598.1)), Paenibacillus sanguinis (WP_018751256.1), Desulfosporosinus sp. Tol-M (KGP77159.1), Thermoanaerobacterium thermosaccharolyticum (WP_013297045.1), Bacillus cereus (WP_001993072.1), Clostridium ljungdahlii (WP_013239896.1), Escherichia coli (WP_001295325.1), Homo sapiens (NP_004784.2), Saccharomyces cerevisiae (NP_009531.1), Haloferax volcanii (WP_004044154.1), Thermococcus kodakarensis (WP_011250215.1), Thermoplasma acidophilum (WP_010901491.1), and Methanocaldococcus jannaschii (AAB99427.1). [file 5759765.f1.pdf]

Supplementary Table 1. Percent sequence identity matrix of Lon-like and Lon proteases from *Methanobrevibacter smithii* (msm_1754 (WP_011954752) and msm_1569 (WP_011954598.1)), *Paenibacillus sanguinis* (WP_018751256.1), *Desulfosporosinus sp.* Tol-M (KGP77159.1), *Thermoanaerobacterium thermosaccharolyticum* (WP_013297045.1), *Bacillus cereus* (WP_001993072.1), *Clostridium ljungdahlii* (WP_013239896.1), *Escherichia coli* (WP_001295325.1), *Homo sapiens* (NP_004784.2), *Saccharomyces cerevisiae* (NP_009531.1), *Haloferax volcanii* (WP_004044154.1), *Thermococcus kodakarensis* (WP_011250215.1), *Thermoplasma acidophilum* (WP_010901491.1), and *Methanocaldococcus jannaschii* (AAB99427.1).

	1	2	3	4	5	6	7	8	9	10	11	12	13	14
Lon-like-Ms (msm_1754) (1)	100.00	64.96	64.22	63.26	62.67	62.67	19.31	18.64	17.38	17.00	13.05	15.67	14.92	17.47
P. sanguinis (2)	64.96	100.00	86.59	74.70	72.19	70.71	19.00	18.68	18.84	15.86	13.94	15.16	15.38	18.73
D. sp. Tol-M (3)	64.22	86.59	100.00	73.52	70.12	70.56	17.71	18.47	17.93	15.03	14.12	13.95	14.97	17.30
T. thermosaccharolyticum (4)	63.26	74.70	73.52	100.00	81.66	83.43	20.44	20.11	19.00	17.42	13.82	14.86	15.29	18.90
B. cereus (5)	62.67	72.19	70.12	81.66	100.00	81.51	20.44	20.29	19.35	17.21	14.63	16.27	15.88	18.29
C. ljungdahlii (6)	62.67	70.71	70.56	83.43	81.51	100.00	19.89	20.11	19.35	17.62	13.62	15.46	14.71	18.29
E. coli (7)	19.31	19.00	17.71	20.44	20.44	19.89	100.00	36.92	37.29	19.15	21.61	21.28	19.11	22.37
H. sapiens (8)	18.64	18.68	18.47	20.11	20.29	20.11	36.92	100.00	44.50	18.28	21.18	19.86	19.56	20.61
S. cerevisiae (9)	17.38	18.84	17.93	19.00	19.35	19.35	37.29	44.50	100.00	19.50	20.44	20.34	21.39	20.82
Lon-Ms (msm_1569) (10)	17.00	15.86	15.03	17.42	17.21	17.62	19.15	18.28	19.50	100.00	50.46	53.07	35.94	57.84
H. volcanii (11)	13.05	13.94	14.12	13.82	14.63	13.62	21.61	21.18	20.44	50.46	100.00	53.00	34.66	55.68
T. acidophilum (12)	15.67	15.16	13.95	14.86	16.27	15.46	21.28	19.86	20.34	53.07	53.00	100.00	35.25	59.65
M. jannaschii (13)	14.92	15.38	14.97	15.29	15.88	14.71	19.11	19.56	21.39	35.94	34.66	35.25	100.00	38.69
M. jannaschii (14)	17.47	18.73	17.30	18.90	18.29	18.29	22.37	20.61	20.82	57.84	55.68	59.65	38.69	100.00

Supplementary Fig. 1

A

B

Lon-like-Ms

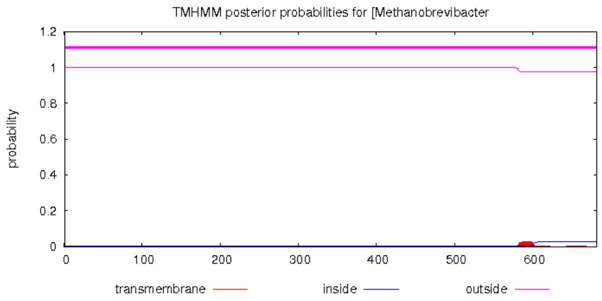

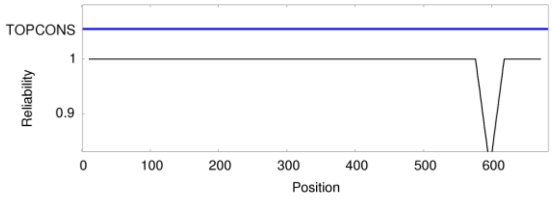

Lon-Tk

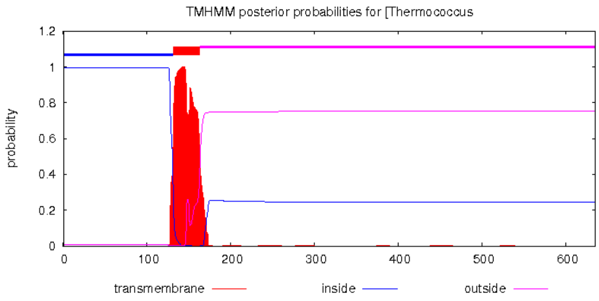

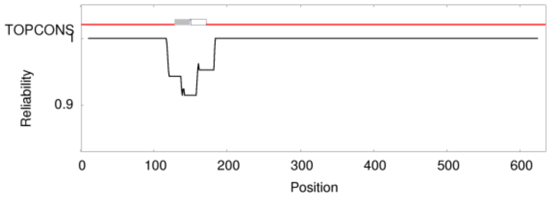

Lon-Ec

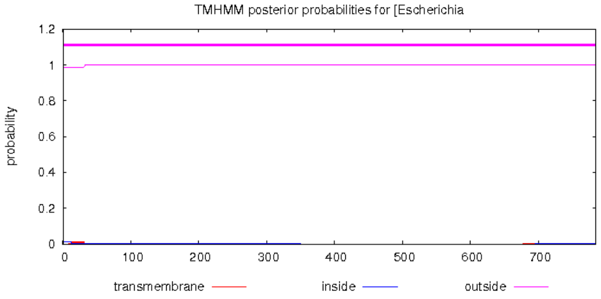

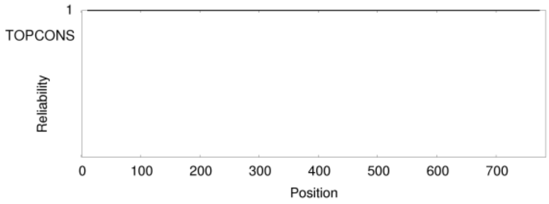

Supplementary Fig. 1. Transmembrane prediction of of Lon-like-Ms, Lon-Ec and Lon-Tk by TMHMM (A) and TOPCONS (B).
